# Supplementary material for: The composition and structure of the outer kinetochore KMN complex is conserved across kingdoms
Source: Commun Biol. 2025 Nov 7;8:1543. doi: 10.1038/s42003-025-09120-6 (PMC12595034; doi:10.1038/s42003-025-09120-6)
Supplement: Supplementary file 1 — Supplementary Information [file 42003_2025_9120_MOESM1_ESM.pdf]

Supplementary figures

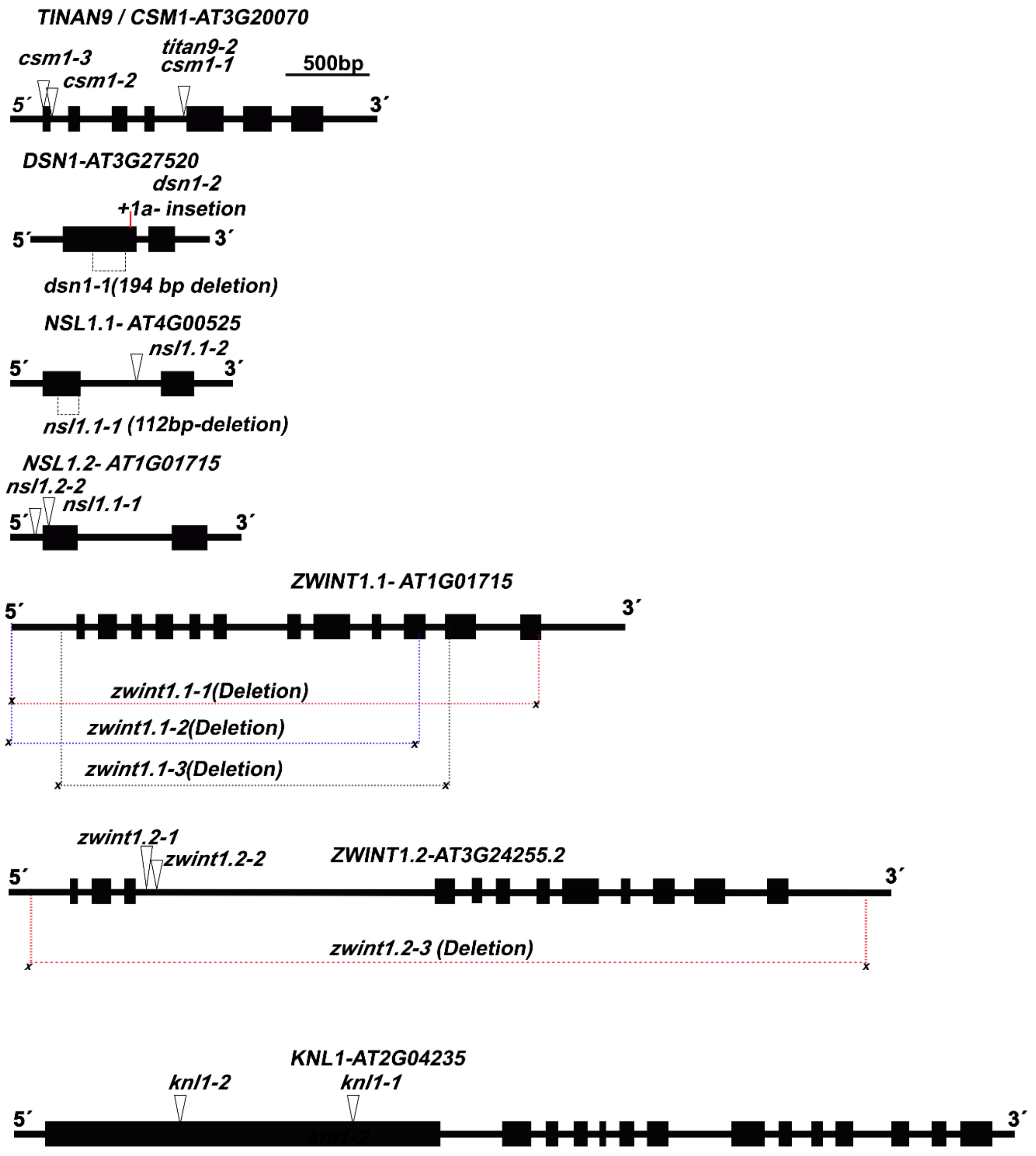

Figure S1. This schematic representation illustrates the Arabidopsis genes *CSM1,* *DSN1, NSL1.1, NSL1.2, ZWINT1.1 ZWINT.2,* and *KNL1*. Each gene is oriented from 5' to 3', as indicated by a horizontal line. Exons are depicted as solid black boxes, while introns and untranslated regions (UTRs) are represented by a black line. Inverted triangles mark the T-DNA insertion points, red vertical lines indicating single base pair insertion in DSN1 and dotted lines (black, blue, red) showing the deleted regions. The name of each gene is presented above the schematic, along with the names of the corresponding mutant alleles.

```
T-COFFEE, Version_11.00 (Version_11.00)
Cedric Notredame
SCORE=99
*
 BAD AVG GOOD
*
ZWINT1.1     :  99
ZWINT1.2     :  99
cons         :   9

ZWINT1.1     MEEETHDGSLDLQEIRRRVKELDFFPRNCREEPVESCSSDYETLVVQDFVLQFEPKVKEIVEEYGDVDLLDV
ZWINT1.2     MEEETHDGSLDLQEIRRRVKEFDFFPRNCREEPVESCSSDYETLVVQDFVLQFEPKVKEIVEDYGDVDLLDV

cons         *********************:****************************************:*********


ZWINT1.1     ED-------SDAYLEYLRNELQSVEAESAKVSEEIERLSQSHAQDSSRLQRDLEGLLLSLDSMSSQDVEKSK
ZWINT1.2     DHTLVDGNLTDAYLEYLRNELQSVEAESAKVSEEIERLSQSHALDSSRLQRDLEGLLLSLDSMSSQDVEKSK

cons         :.       :********************************* ****************************


ZWINT1.1     ENQPSSSSMEVCEVIDDDKFKMFELENQMEEKRMILKSLEDLDSLRKRFDAAEQVEDALTGLKVLEFDGNFI
ZWINT1.2     ENQPSSSSMEVCEVIDDDKFKMFELENQMEEKRMILKSLEDLDSLRKRFDAAEQVEDALTGLKVLEFDGNFI

cons         ************************************************************************


ZWINT1.1     RLQLRTYIQKLDGFLGQHKFDHITEPSELIHELLIYLKDKTTEITKFEMFPNDIYIGDIIEAADSFRQVRLH
ZWINT1.2     RLQLRTYIQKLDGFLGQHKFDHITEPSELIHELLIYLKDKTTEITKFEMFPNDIYIGDIIEAADSFRQVRLH

cons         ************************************************************************


ZWINT1.1     SAVLDTRSSVQWVVAKVQDKIISTTLRKYIVMSSKTIRYTFEYYDKDETIVAHIAGGIDAFLKVSDGWPLLN
ZWINT1.2     SAVLDTRSSVQWVVAKVQDKIISTTLRKDFVMSSKTIRYTFEYYDKDETIVAHIAGGIDAFLKVSDGWPLLN

cons         **************************** :******************************************


ZWINT1.1     TPLKLASLKNSDNQSKGISLSLICKVEELANSLDLETRQNLSGFMDAIEKILVEQTREELQSNKSSQK
ZWINT1.2     TPLKLASLKNSDNQSKGFSLSLISKLEELANSLDLETRQNLSGFMDAVEKILVQQTREELKSNESSQK

cons         *****************:*****.*:*********************:*****:******:**:****
```

Figure S2. Multiple sequence alignment of two paralogs of ZWINT1 proteins of Arabidopsis KMN complex. The sequences have been aligned using T-coffee and shows 95% protein identity.

```
T-COFFEE, Version_11.00 (Version_11.00)
Cedric Notredame
SCORE=84
*
 BAD AVG GOOD
*
NSL1.1       :  84
NSL1.2       :  84
cons         :   8

NSL1.1       MESMASDPELD--MDEGDLEKLESDVKQMAKKISEYRQTLPDHLRNTLDSALSSHIPVFPNIDSGSDPLPSS
NSL1.2       MAFEHRNEDLNSLDSEEDLEKMESEVEQMSRKVSEYRKSLPSNLSNS---VISSRRSNFPNIDSLGFRTTST

cons         *     : :*:   .* ****:**:*:**::*:****::**.:* *:   .:**: . ****** .   .*:


NSL1.1       CLTIAE-AQVPGVLEEQDSEEKWIQLKERMSRNAANIPKVVKRMRECIESIDKLDSLE-VTIHPAFKRQRIN
NSL1.2       ALVSRDGAHIGGI--EQESRDSLIQLKERVYENIATVPLVVERMRESKERIDKSISFNGTTMHPAFTRRKAS

cons         .*.  : *:: *:  **:*.:. ******: .* *.:* **:****. * ***  *:: .*:****.*:: .
```

Figure S3. Multiple sequence alignment of two paralogs of NSL1 proteins of Arabidopsis KMN complex. The sequences have been aligned using T-coffee and shows 43% protein identity.

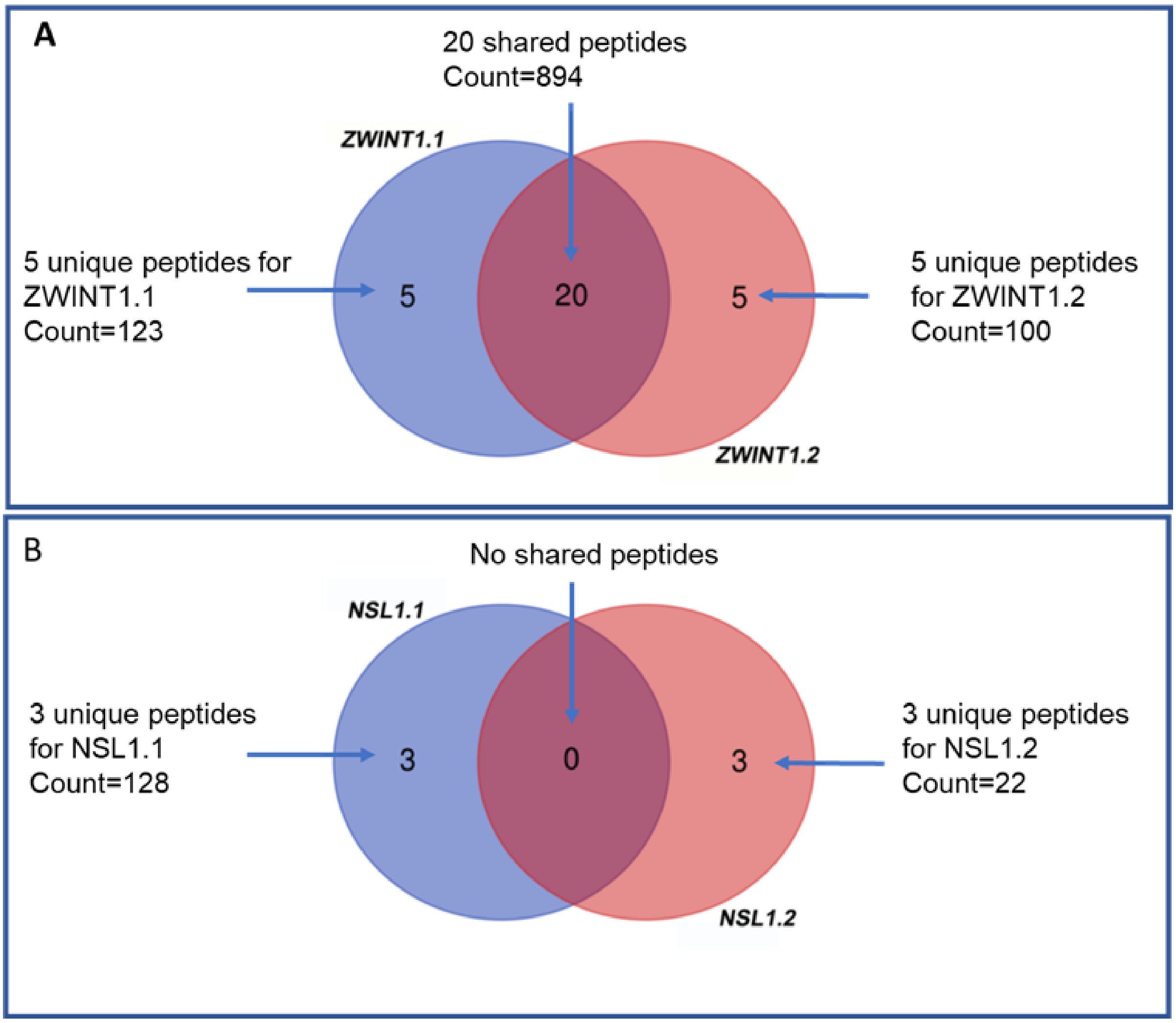

Figure S4 Venn diagrams illustrating the distribution of unique and shared peptides between two paralogs of two proteins the paralogs ZWINT1 and NSL1 in AP-MS analyses.

(A) Peptide analysis from all five AP-MS experiments shows 20 shared peptides with a total spectral count of 894. Additionally, ZWINT1.1 has 5 unique peptides with a count of 123, while ZWINT1.2 has 5 unique peptides with a count of 100. (B) Peptide analysis from NSL1 AP-MS show that NSL1.1 has three unique peptides with a total spectral count of 128, while NSL1.2 also has three unique peptides with a total spectral count of 22. No shared peptides were detected between NSL1.1 and NSL1.2.

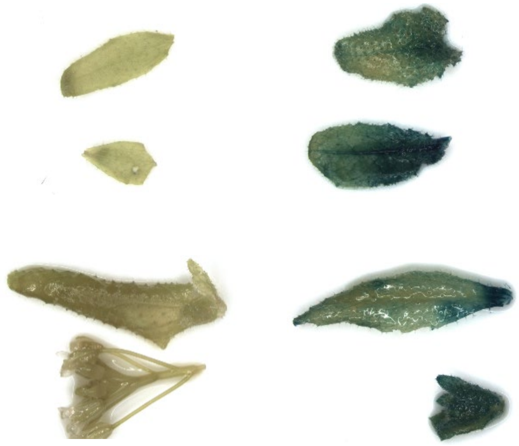

Figure S5.- GUS Expression in Venus GUS Transgenic vs. Wild-Type Control.

The Venus GUS control transgenic line (on the right) shows GUS expression in Cauline leaves and actively dividing tissue, whereas the wild-type control tissue (on the left) exhibits no GUS expression

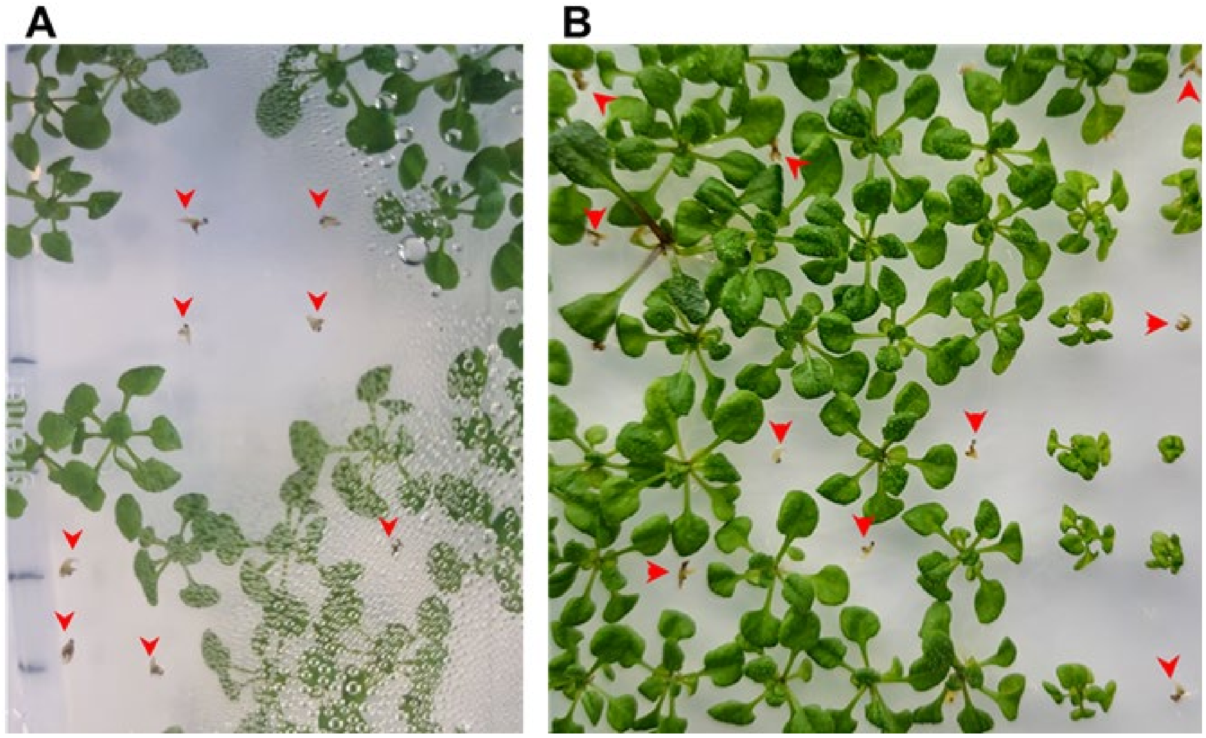

Figure S6.- Seeding progeny of *zwint1.1-1* (-/+) and *zwint1.2-3* (-/+).

(A) Progeny of *zwint1.1-1* (+/–) and (B) progeny of *zwint1.2-3* (+/–) plants were sown on MS agar medium. While most seedlings appear normal, a proportion display seedling lethality shortly after germination (indicated by red arrowheads).

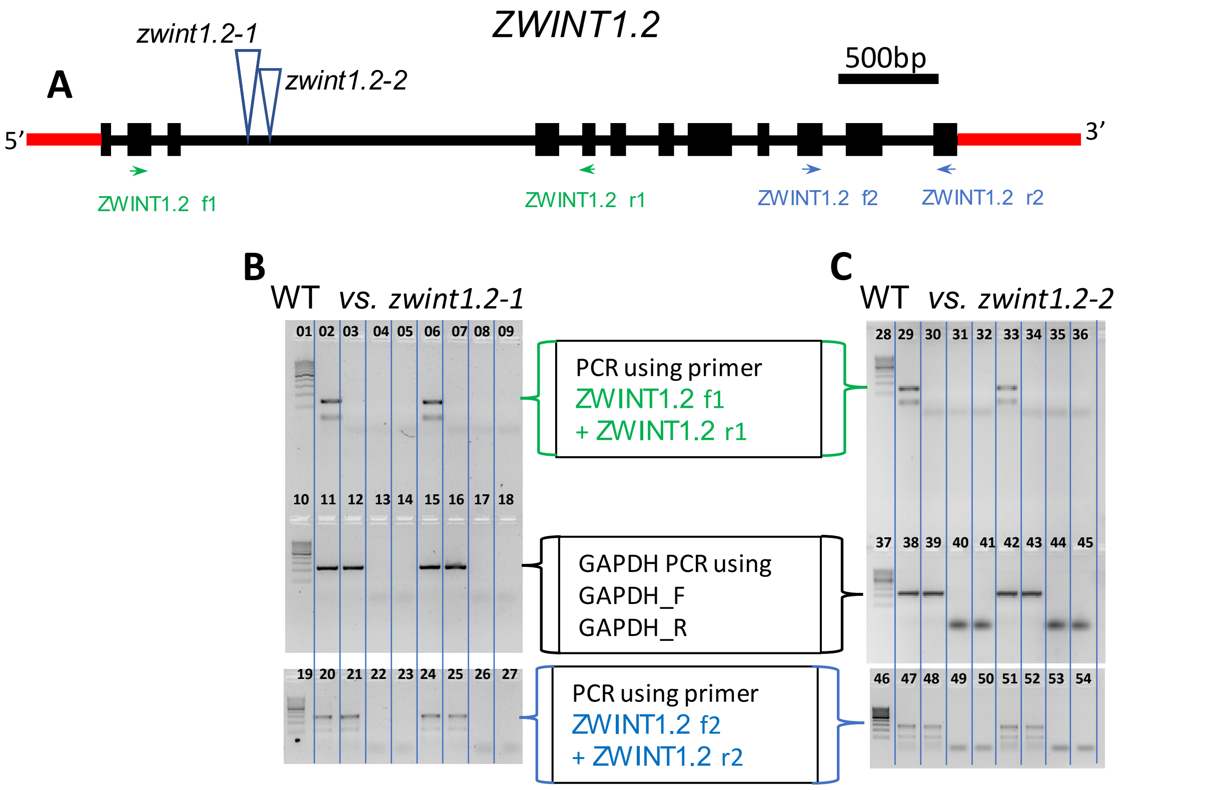

**Well no.**	**Template name**
1,10,19	100bp ladder
2,11,20	WT (RT) replicate1
3,12,21	*zwint1.2-1*(RT) replicate1
4,13,22	WT (-RT) replicate1
5,14,23	*zwint1.2-1* (-RT) replicate1
6,15,24	WT (RT) replicate2
7,16,25	*zwint1.2-1* (RT) replicate2
8,17,26	WT (-RT) replicate2
9,18,27	*zwint1.2-1* (-RT) replicate2

**Well no.**	**Template name**
28,37,46	100bp ladder
29,38,47	WT (RT) replicate1
30,39,48	*zwint1.2-2* (RT) replicate1
31,40,49	WT (-RT) replicate1
32,41,50	*zwint1.2-2* (-RT) replicate1
33,42,51	WT (RT) replicate2
34,43,52	*zwint1.2-2* (RT) replicate2
35,44,53	WT (-RT) replicate2
36,45,54	*zwint1.2-2* (-RT) replicate2

Figure S7. Expression analysis in *zwint1.2* mutants.

A. Schematic representation of the *ZWINT1.2* gene. Black boxes indicate exons, black lines denote introns, and red lines represent untranslated regions (UTRs). Triangles mark the T-DNA insertion sites in *zwint1.2-1* and *zwint1.2-2*. **B & C**. Agarose gels showing RT-PCR results from 10-day-old sister wild-type (WT), compared to *zwint1.2-1*, and *zwint1.2-2, respectively*. "+RT" indicates cDNA synthesis with reverse transcriptase, while "–RT" serves as a control for genomic DNA contamination. Each experiment was performed with two independent biological replicates. RT-PCR of the GAPDH gene is used as a positive control.

In contrast to the wild type, both *zwint1.2-1* and *zwint1.2-2* show no detectable expression with the f1/r1 primer set (Pannel B: wells 3 and 7 vs. 2 and 6; C: wells 30 and 34 vs. 29 and 33). However, both mutants exhibited comparable expression to the wild type when tested with the f2/r2 primer set (B: wells 21 and 25 vs. 20 and 24; C: wells 48 and 52 vs. 47 and 51). Thus for both mutants, a full length protein cannot be expressed, but a truncated protein (e.g. with M135 as translation initiation codon) might be expressed.

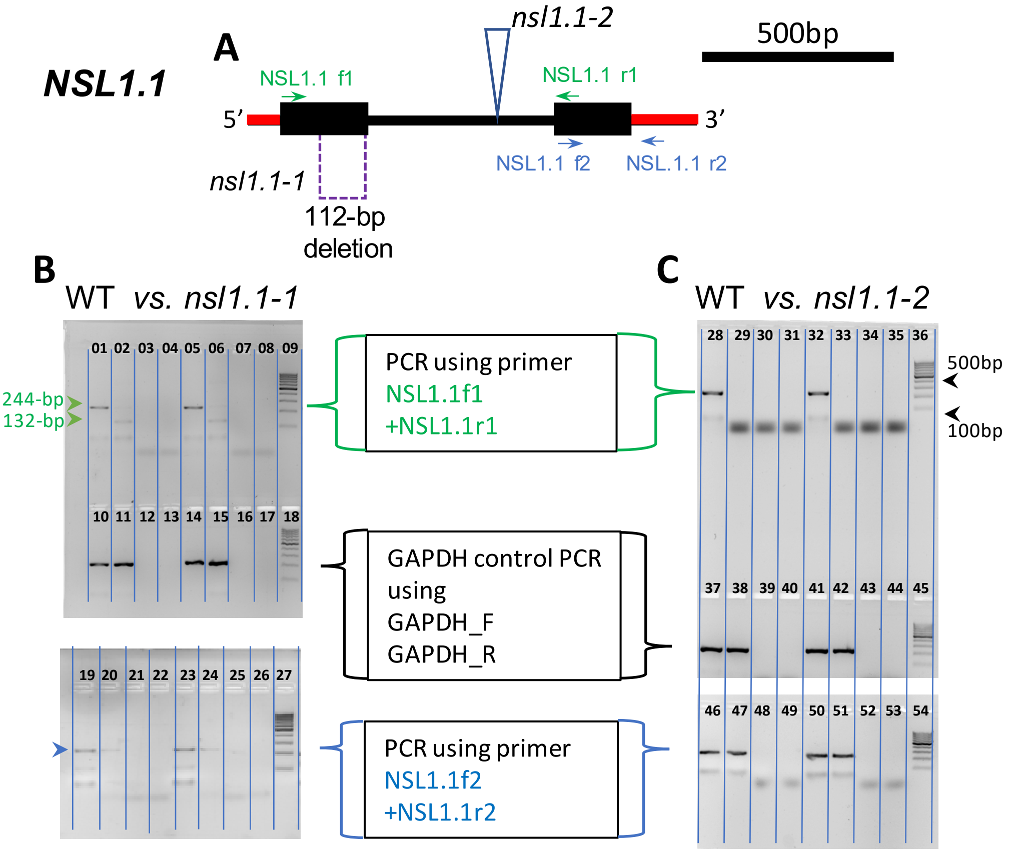

**Well no.**	**Template name**
1,10,19	WT (RT) replicate1
2,11,20	*nsl1.1-1*(RT) replicate1
3,12,21	WT (-RT) replicate1
4,13,22	*nsl1.1-1*(-RT) replicate1
5,14,23	WT (RT) replicate2
6,15,24	*nsl1.1-1*(RT) replicate2
7,16,25	WT (-RT) replicate2
8,17,26	*nsl1.1-1*(-RT) replicate2
9,18,27	100bp ladder

**Well no.**	**Template name**
28,37,46	WT (RT) replicate1
29,38,47	WT (RT) replicate1
30,39,48	*WT (-RT) replicate1*
31,40,49	*nsl1.1-2* (-RT) replicate1
32,41,50	*WT (RT) replicate2*
33,42,51	nsl1.1-2 (RT) replicate2
34,43,52	*WT (-RT) replicate2*
35,44,53	nsl1.1-2(-RT) replicate2
36,45,54	*100bp ladder*

Figure S8. Expression analysis of *nsl1.1* mutants.
A. Schematic representation of the *NSL1.1* gene. Black boxes represent exons, black lines represent introns and red lines indicate untranslated regions (UTRs). The dotted purple box indicates the CRISPR-Cas9 mediated deleted region in *nsl1.1-1*. The triangle mark the T-DNA insertion site in *nsl1.1-2*. Green and blue arrows indicate PCR primers. **B & C** Agarose gels show RT-PCR results from 10-day-old sister wild-type (WT) seedlings compared with *nsl1.1-1* and *nsl1.1-2,* respectively. “+RT” denotes cDNA synthesis using reverse transcriptase, while “–RT” serves as a control for genomic DNA contamination. Each experiment was performed with two independent biological replicates. RT-PCR of the GAPDH gene is used as a positive control.
RT-PCRs with the f1 and r1 primers indicates that *nsl1.1-1* produces a shorter transcript compared to wild-type (Pannel B, wells 2 and 6 *vs* 1 and 5), and suggests a lower expression level. Consistently, f2/r2 RT-PCRs in *nsl1.1-1* suggest a lower expression level of the truncated *NSL1* compared to wild-type (panel B, wells 20 and 24 *vs* 19 and 23). As the *nsl1.1-1* deletion leads to a frameshift, the expressed gene is unlikely to produce a functional protein.
In *nsl1.1-2,* RT-PCRs with the f1 and r1 primers failed to amplify the *NLS1* transcript, indicating that the T-DNA prevent expression and/or splicing (panel C, wells 29 and 33 vs. 28 and 32). In contrast, f2/r2 RT-PCRs suggested a similar level of expression than wild-type. (Figure X, C, wells 47 and 51 vs. 46 and 50. This suggests either the presence of an unspliced mRNA or that the T-DNA serves as promotor of a truncated genes. In both cases, it is unlikely that *nsl1.1-2* produces a functional protein.

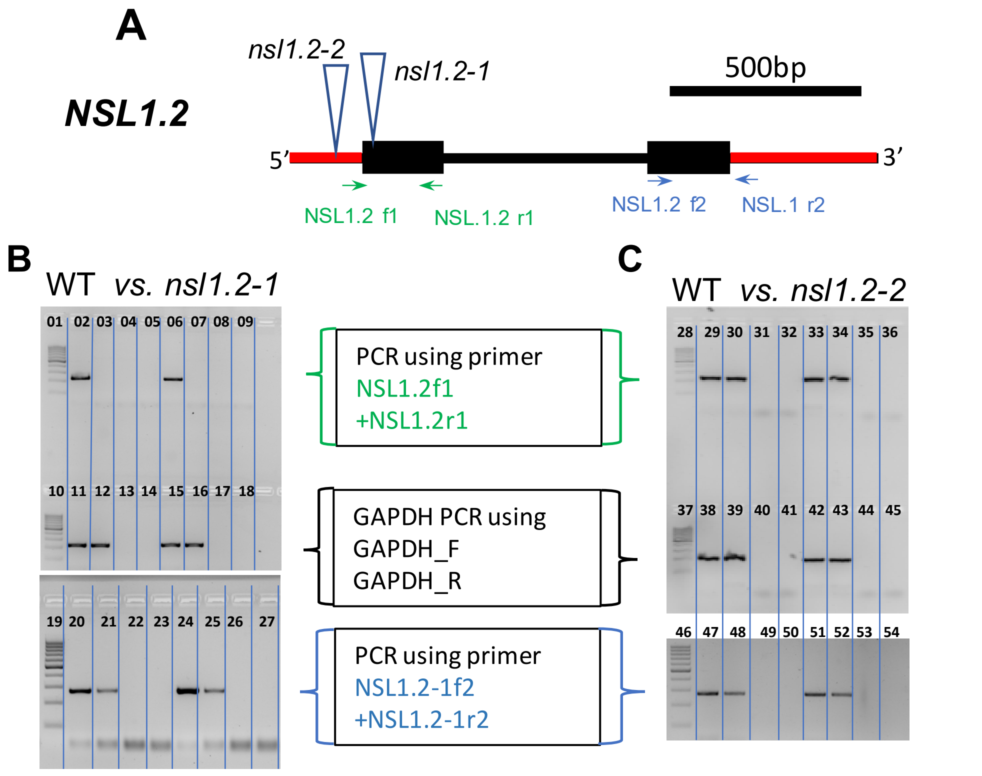

Well no.	Template name
1,10,19	100bp ladder
2,11,20	WT (RT) replicate1
3,12,21	*nsl1.2-1*(RT) replicate1
4,13,22	WT (-RT) replicate1
5,14,23	*nsl1.1-1*(-RT) replicate1
6,15,24	WT (RT) replicate2
7,16,25	*nsl1.2-1*(RT) replicate2
8,17,26	WT (-RT) replicate2
9,18,27	*nsl1.2-1*(-RT) replicate2

Well no.	Template name
28,37,46	100bp ladder
29,38,47	WT (RT) replicate1
30,39,48	*nsl1.2-2*(RT) replicate1
31,40,49	WT (-RT) replicate1
32,41,50	*nsl1.2-2*(-RT) replicate1
33,42,51	WT (RT) replicate2
34,43,52	*nsl1.2-2*(RT) replicate2
35,44,53	WT (-RT) replicate2
36,45,54	*nsl1-1*(-RT) replicate2

Figure S9. Expression analysis of *nsl1.2* mutants.
A. Schematic representation of the *NSL1.2* gene. Black boxes represent exons, black lines represent introns and red lines indicate untranslated regions (UTRs). The two triangles mark the T-DNA insertion sites in *nsl1.2-1* and *nsl1.2-2*. Green and blue arrows indicate PCR primers. **B & C.** Agarose gels show RT-PCR results from 10-day-old sister wild-type (WT) seedlings compared with *nsl1.2-1 and nsl1.2-2* mutants, respectively. “+RT” indicates cDNA synthesis with reverse transcriptase, while “–RT” serves as a control for genomic DNA contamination. Each experiment was performed with two independent biological replicates. RT-PCR of the GAPDH gene is used as a positive control.

RT-PCRs with the f1 and r1 primers detected the *NSL1.2* mRNA in the wild type but not in the *nsl1.2-1* mutant (B, wells 3 and 7 vs. 2 and 6). Note that the T-DNA insertion may prevent the amplification of a long mRNA. RT-PCR with the f2/r2 set of primers detected expression in *nsl1.2-1,* but suggested a lower level than in the wild type (panel B, wells 21 and 25 vs. 20 and 24). The *nsl1.2-1* mutant thus express an mRNA that could encode a truncated protein with the M29 serving as translation initiation codon.

The *nsl1.2-2* mutant displayed expression levels similar to wild-type with both primer sets (Figure X, C, wells 29 and 33 vs. 28 and 32; wells 48 and 52 vs. 47 and 51). It is thus likely that the *NSL1.2* gene is functional in *nsl1.2-2*.

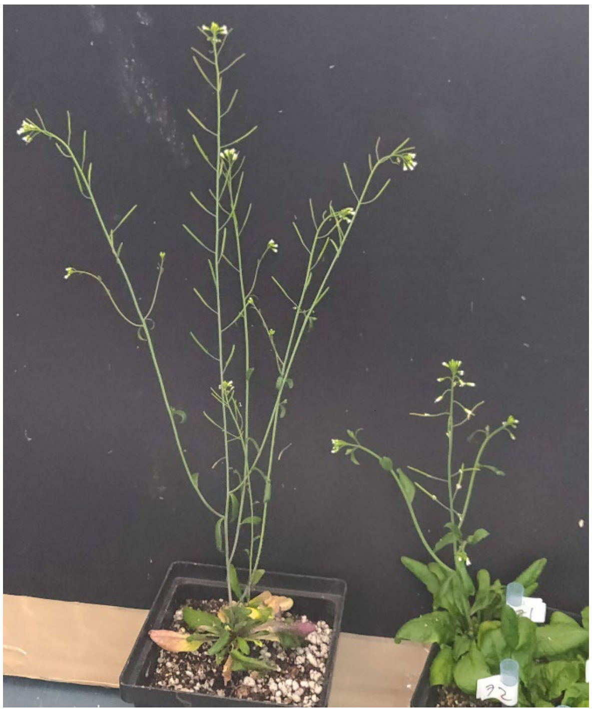

Figure S10.- The *nsl1* double mutant demonstrates slow growth.

The *nsl1.1-1 (-/-) nsl1.2-1* (-/-) double mutant (on the right) exhibits slower growth compared to the *nsl1.1*(-/-) single mutant (on the left).

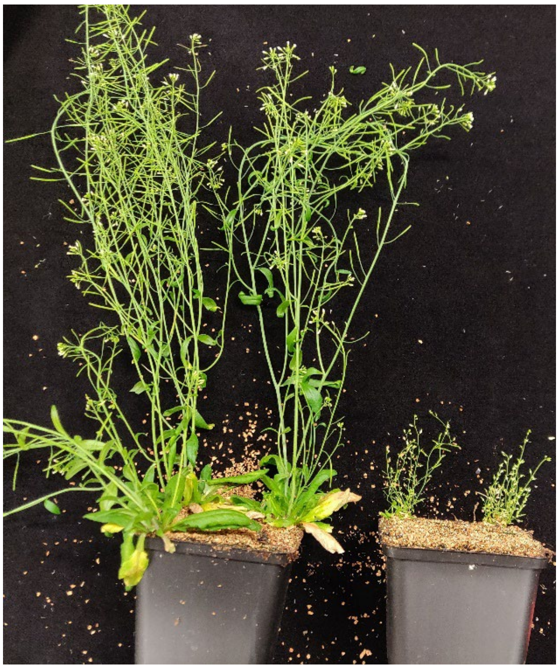

Figure S11.- The *mutant esd4* demonstrates slow growth.

The *esd4* mutant (on the right) exhibits developmental growth defects compared to the wild type *and* heterozygous sister plant (on the left).

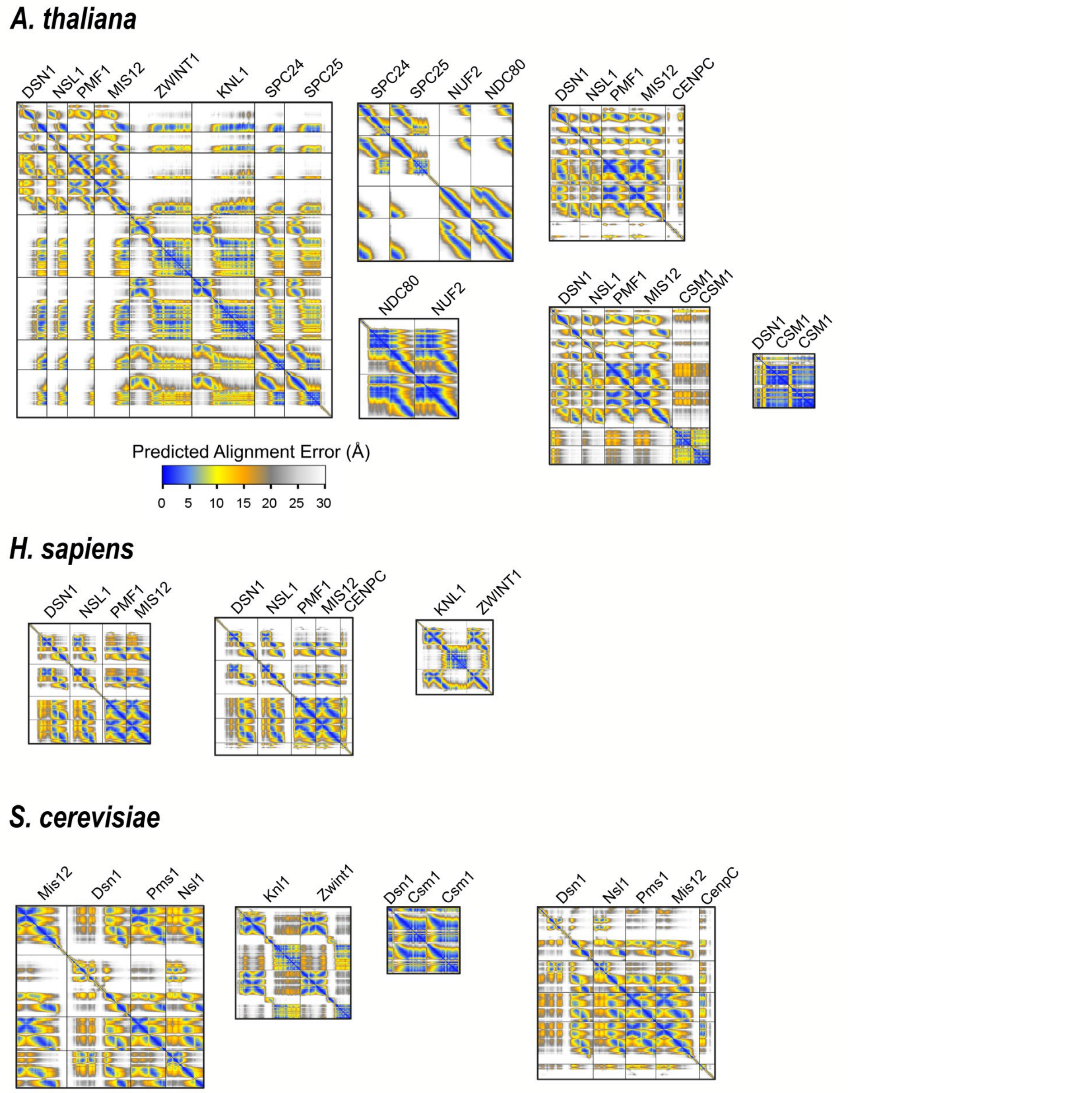

Figure S12.- Predicted Alignment Error (PAE) maps of the 13 generated models

Predicted Alignment Error (PAE) maps generated by AlphaFold3 and displayed using ChimeraX for the 13 models generated in this study (6 in *A. thaliana*, 3 in human and 4 in yeast). The associated confidence scores are provided is Source data set 5.
