# Supplementary material for: The composition and structure of the outer kinetochore KMN complex is conserved across kingdoms
Source: Commun Biol. 2025 Nov 7;8:1543. doi: 10.1038/s42003-025-09120-6 (PMC12595034; doi:10.1038/s42003-025-09120-6)
Supplement: Supplementary file 2 — Supplementary Data 1–5 [file 42003_2025_9120_MOESM2_ESM.zip › Source data set/Source data set3.docx]

**Source data set 3**

**AT3G27520-*DSN1***

**Full length genomic sequence**

taggaaagctgatatggtaaactgaagcacgttgatttgtatgaatgaaaatgttttctattgatgataattaaaatgagatgtcaacttgaaaaaaccatactgtattgattcctattttaatatttgttagtctttgtttaatttaaacgctaatatcgaagtttggcctgccatgttagttagtttactttgacttattcatttttgtatctttgctcttctaagaactctcaatagacaagttttctttctttctttttaattaaataatagaaaatcattacactgaagataaaa

gaaatatttatttcagcagttgtatgaatcttgatcattgaaggaagaagtcaatgtcatgacagctaacattgagccgtcgataacacattcgagattagtcattaaataaagaccatggtgtgtgctataataatgttaagaacggtattaaacggtgtgcttaataattggcttttttgtgggttaacttatattttacacgaatatactattgggccttaagtatttagttatcggcccataagcatttaacgataacgggagtttggtgaatccgaagctaatctctaaaatctt

tcagtttcgagatccaaagtcactcacttttttctgaaattcgctactcagaattttcccgctccactgcatctctacggcgaATGGACTCACCGATGTCAACGAGTTCCATAGACGGAGACGAAGATTGGGAATGTTACGAAGACGATGGCTTCGTTTACAATCGAAAAAAGCGAACCCGTTTCGCCGACGCTGAAGAAACATCAAAACCGCCGGATCCGGAGTTAGATCGGGTGGTAGAAGAGAGGAATCGAAGGATACGGAAGAAGAGATTACTGGTGAAGCTCAAAAGAAAGTA**CC**

**AGAGTGAGATCGACCAG**TGGGAGATTTTATCGAATAGTTTCAATGCTATGCAAGAGAAAGCAGATCGATTTCAAACGGCGCAAAGAGAAGAGAGGTTAAATGCGAACGAAACGATGTCGTTTCCTGGGAATTCTTCCTCTACAACAGAAGGAGGCCGAGAATTTGGCGGAGAAGATGCTTCTAAGTCGCCG**TCGTCTATGCTCGACCAAC**TTCTTTTTACGgtaattagggtttggactcaattcgatgaaagttttgaaattggttcgtttttgaaatttgaattttgaaattttgcag

GTGGAAAGACAAGAAGCGGTAATCAACGAAGTCTCAAAGCTATGTGAAGTTACAGAGAACATATGTAGAGTGGAAGAAGAAGAAACGAAGCAGTCCTTCTTTGATCTTCCCATATGGAGCTCACCAACGGATCTAATGGCATCGCTTTGTGGTGATTAAcacttaaattagtcctcttatgtggtttatctgagctctctacttatgtttactttgtacaatgcgtgtgtgttgtgttttgttgcaattgtagagtgattttgtgtgttaggctcgaattagggggtgttctatccggat

Tttagtcagttatatcctcaattttcaaaaaaagcttcagttttatactggtttttccggtacggtttcgggttcggggtttggatatagatatgggttcaggtaagtttgtcttagattgaatccttcctatgctcaagaattatgtatctatttttgccatacctcggattctataaatcatgaatattttgaaggcaaacactaattgaactgagtacttgtttgattgtttcaacaaaatatgaaaagcataacaacctgcagtaacttaaaaccacattgtgtttcattgttaag

nnnnnn- genomic sequence upstream on annotated 5’ utr

nnnnnn- 5’and 3’utr

NNNNNN- Exon

nnnnn- intron

Nnnnn- deleted sequence

ATG- start codon

NNN- stop codon

**Nnnnnn**(Bold and underlinelined)- guide RNA target recognition sequence

***dsn1-1***

**deleted sequence**

CAGTGGGAGATTTTATCGAATAGTTTCAATGCTATGCAAGAGAAAGCAGATCGATTTCAAACGGCGCAAAGAGAAGAGAGGTTAAATGCGAACGAAACGATGTCGTTTCCTGGGAATTCTTCCTCTACAACAGAAGGAGGCCGAGAATTTGGCGGAGAAGATGCTTCTAAGTCGCCGTCGTCTATGCTCGACCA

***dsn1-2***

***a-Insertion of an adenine***

TCTAAGTCGCCGTCGaTCTATGCTCGACCAAC

Dsn1-2

position chr3: 110192895 insertion of an adenine (a) in the Columbia (TAIR10) genome.

***NSL1.1***

**AT4G00525 full length genomic**

gaaagggagaggtacgttgttgcatcgaagaagaatgaagaatctaatgtggtttgcaaaaatcgagctcttattcttttcaaacgttaatatatagtttgataatttgtgtgcagctggtggtatcattgacgcaagaagctgtgttaagagaagctgtgactattaagccatccttcggggccaagctatgaagccataggattttgatagtgagagaattgctgcatctgttactcctcacggtcacattccaaagagtcgtcacttatactacatttgcatatgtttttcgatccacaattattattttcccctctaaaagggtctacacatatgtttgtttgtaaccacgataatgtttcaacagcaatgaaaaagcaaacagtggttcattttacaggttaacgacgtcgttttggatacaccctcttttaacgttattatcattttatcacacagtcccagtcttcagagttaaagacgctcctcaattttgaaaaaaccctccatagtatctcttctacagaaccttgagttttcggagaacctcgaatctcaATGGAATCAATGGCTTCAGATCCCGAACTTGACATGGATGAAGGAGATCTGGAGAAATTGGAGTCAGATGTGAAGCAGATGGCCAAGAAGATCTC**CGAGTACAGGCAAACCCTAC**CGGATCATCTCAGGAACACTCTTGATTCTGCGCTATCTTCCCACATACCCGTTTTCCCCAACATCGATTCCGGGTCGGATCCTCTTCCGTCGT**CATGCCTCACTATCGCAGgc**aggttccttctttgttccttttttttttggtttttgcgaaatcactttttggttagagaaattataagcaggtctaaatttagggcttattcctggaaattatgtgtcttttctcttcgtgagaattggtgatgaatcaactacactcactagaacttgtcaaggttaaagctaaaagctagagtcttttttgtagttttgaagatcttgtcaagtattcacagtatcgtagagaaacataagcttgcagtgattcttttcttcttcctctcttatcagtatgatatatcaatcaatctgctgtgttttatatttaatgcagaaaataggcgcataataatgttgtagttcataagagactttgcttgagaagagttcatagtattgtagagatacacaagagcctgcactgatctttgtcatcttcccctcatatcagtttgctgtattaaccaatcttcggtgtttttcttttaaatgcagAAGCACAGGTACCTGGTGTGTTGGAAGAGCAAGACTCTGAAGAGAAATGGATTCAGCTCAAGGAAAGAATGTCGAGGAACGCTGCGAATATACCTAAAGTTGTAAAGAGAATGCGAGAATGTATAGAGAGTATCGACAAACTCGATTCTTTGGAAGTTACCATTCACCCGGCATTCAAAAGGCAAAGAATTAACTGActatttctccttcatgcttaggctctccatagtattttcttaggaaagatatgtgtgctgatttgtgaatatgtaattctagattgatctggcatgtacattggtgtcgtttgattcaaaacattcacaacatgatatttgattgtgcaagtactagggaagagattaagacttgttactggaaacaaagtcaaagtaaagggttaaaccatagttgttgatcacaaaagt

*nsl1.1-1* **deleted sequence**

**TAC**CGGATCATCTCAGGAACACTCTTGATTCTGCGCTATCTTCCCACATACCCGTTTTCCCCAACATCGATTCCGGGTCGGATCCTCTTCCGTCGT**CATGCCTCACTATCGC**

*NSL1.1* CDS- protein coding sequence

ATGGAATCAATGGCTTCAGATCCCGAACTTGACATGGATGAAGGAGATCTGGAGAAATTGGAGTCAGATGTGAAGCAGATGGCCAAGAAGATCTCCGAGTACAGGCAAACCCTACCGGATCATCTCAGGAACACTCTTGATTCTGCGCTATCTTCCCACATACCCGTTTTCCCCAACATCGATTCCGGGTCGGATCCTCTTCCGTCGTCATGCCTCACTATCGCAGAAGCACAGGTACCTGGTGTGTTGGAAGAGCAAGACTCTGAAGAGAAATGGATTCAGCTCAAGGAAAGAATGTCGAGGAACGCTGCGAATATACCTAAAGTTGTAAAGAGAATGCGAGAATGTATAGAGAGTATCGACAAACTCGATTCTTTGGAAGTTACCATTCACCCGGCATTCAAAAGGCAAAGAATTAACTGA

*nsl1.1-1* putative CDS- protein coding sequence

ATGGAATCAATGGCTTCAGATCCCGAACTTGACATGGATGAAGGAGATCTGGAGAAATTGGAGTCAGATGTGAAGCAGATGGCCAAGAAGATCTCCGAGTACAGGCAAACCCAGAAGCACAGGTACCTGGTGTGTTGGAAGAGCAAGACTCTGAAGAGAAATGGATTCAGCTCAAGGAAAGAATGTCGAGGAACGCTGCGAATATACCTAAAGTTGTAAAGAGAATGCGAGAATGTATAGAGAGTATCGACAAACTCGATTCTTTGGAAGTTACCATTCACCCGGCATTCAAAAGGCAAAGAATTAACTGA

NSL1A

MESMASDPELDMDEGDLEKLESDVKQMAKKISEYRQTLPDHLRNTLDSALSSHIPVFPNI

DSGSDPLPSSCLTIAEAQVPGVLEEQDSEEKWIQLKERMSRNAANIPKVVKRMRECIESI

DKLDSLEVTIHPAFKRQRIN

*nsl1a*-1

MESMASDPELDMDEGDLEKLESDVKQMAKKISEYRQTQKHRYLVCWKSKTLKRNGFSSRKECRGTLRIYLKL

Single letter amino acid code

High heighted amino acids sequences are identical sequence in wild type (NSL1.1) and mutant (nsla-1)

AT3G23910 *ZWINT 1.1*

Full length genomic seq

Aaatattcgatacaatcggtacaataagaataactatatgtaaatatcaaaatctctaccctgaagtaaggaagattttttattggacattttaatgggggcagatttactgaaagataaaataaaataaagcagatgtggctactaga**gggtcgaatttgggagctga**gggaacatactcactgatatataattatgattttgattaaatttaattaagttttttttttctttttttttaaaaggggaagtaaacgttacagattaaaattaaagagtctt**ctggttcaatacgttacgat**tggtcataataaattgaaccggaattgcaattaacttcaaccctctgggtcaaattgattcaaatagaattcaggggttaatgttgaagaaaagtataaacctcggggagagttatgtttctcttccgtttctctttcacctttcccatttgaaaattgaaatcgtcttctctgctttcaacttcacaactacactctcttcagttctctaacagtagcgccaaaatgaatccccaagactaaagctttcttcaATGGAAGAAGAAACCCACGACGGATCTCTCGATCTCCAAGAGATTCGCAGgttttctacttctctttcgtctctttctttcattgatttttgtgctgctttgttcatctatttgttctcatttttctcagGCGCGTGAAAGAGCTCGATTTCTTTCCCCGGAATTGCAGAGAAGAGCCTGTAGAATCGTGTAGTTCCGATTACGAGACTCTGGTGGTTCAAGATTTTGTTCTTCAGTTTGAAgtaattgcgagatttcgagaacccttttccgtttttcaaatttgtctaattttcgattagattgctaatttcgttttcgtgtagCCAAAGGTGAAAGAGATTGTTGAAGAATACGGCGATGTTGATTTATTGGATGTTGAAGATTCAGgtttgctctgttgtttcctgtgagattaagatgaagtatgaaagtcaaataatggtgaaattaagtgatggaaatctgacagATGCTTACTTGGAGTACTTGAGAAATGAGCTTCAGTCTGTGGAGGCTGAAAGTGCCAAAGTTTCTGAAGAAATTGAACGTCTTTCTCAGTCTCATGCCCAAGgttcttcattagttttcttctactgatttagttttagatcatatagagtttgctactgttatttttataatatgagttttcttctatggatttgttagATTCTAGTAGGTTGCAAAGGGATCTTGAAGGTCTTTTACTGTCACTGGATTCTATGTCATCTCAGgttatttggttgaatagattatttctttggctggaatcaaaaggcttagttttgagagttatgtgattcttattttagGATGTGGAGAAGTCAAAAGAGAATCAACCATCTAGCAGCTCAATGGAAGTATGTGAAGTGATTGATGATGACAAGTTTAAGgtgccatgtttttaagttttgtttctaagagaagaagcttttgtgtgtgtgtgtgtgtgtgtgtgaaacatgtattaacaaaatttcatctatcttccagttattgattagagctaatgtggcagaatgtactaacctacttttcccgagcacgctaaggcagaagatcatatactgtcctttcagttataatttctaaataatttcgtagtggttaagacttaacatgaaaaagcatctattgtactctaaaatttccagatgttttttctagtacttactttctttgcttgtcactgcctatcttcttattgcttttatattgtgcatggatttgatcatctttcacatgtttttcagATGTTTGAACTCGAAAATCAGATGGAGGAGAAAAGGATGATTCTTAAGTCATTGGAAGATCTGGATTCGTTACGTAAAAGgtaatttaaacgtactttcagatttacatatttaccctgtcttcattagagttcttcacttgttctaccttcagGTTTGATGCTGCAGAACAAGTTGAGGACGCATTGACAGGCTTGAAGGTGCTCGAGTTTGACGGAAACTTTATTAGGCTCCAACTGCGAACATATATTCAAAAACTAGACGGTTTTCTTGGACAGCACAAATTTGACCACATTACTGAGCCATCTGAATTGATCCATGAATTGCTAATATACCTTAAGGATAAAACTACAGAGATAACAAAATTTGAGgtaagaacactctccgtctttcactgctgactattatgctttagttttgcataaatcctagataaatgtatctgtttcttgcttcttgttcctttttgtgaataaaattgaaatctgctttcttgctcagATGTTTCCAAATGATATATACATAGGAGACATCATCGAGGCAGCTGATTCTTTCAGgtttgcgctctagtatctttatgtcctgaaatttgtattacttatcattctgaaactgtatggtacgtaagttataagactaataagttatcagggacgataagatgatatcattctattattcttaaaactgcagGCAGGTAAGGTTACACTCTGCAGTGCTAGACACAAGATCTTCAGTCCAGTGGGTTGTCGCCAAAGTGCAAGATAAAATTATTTCAACAACTTTGAGAAAATATATTGTGATGAGTTCGAAAACAATTAGgtgagccttccctttcagtagcttgagaggctgaatttttctttcgtagctaactcatgtcattgagtgtctgagcaagccaattgactgcatgttttgtgatcct**atggcagGTACACGTTTGAA**TACTACGACAAAGATGAAACAATTGTGGCTCATATAGCTGGAGGTATTGATGCATTTTTAAAGGTCTCTGATGGTTGGCCACTGCTGAATACCCCATTGAAGCTTGCATCTCTCAAGAACTCTGACAATCAGTCAAAGGGAATTTCTCTGAGCCTTATCTGCAAAGTTGAGgtaagatcatgtttctccaaactcaacactttcttctcgattttacaattccatcataaacaaaggtcttctctgttccttctcctggtcattgttgtcagtcttatttaatctctcaaatcgaacatttaggatatataataacatgattaagccattgtcctagaccaaatacagtttgcaacattgtagatgtaagtgcgtttggttttgctaagcgtaacgatttgtctcttgctctactcgtcagGAACTAGCGAATTCCTTGGATTTAGAGACCCGGCAAAACTTATCAGGCTTCATGGATGCAATTGAGAAAATACTCGTGGAGCAAACCC**GTGAAGAACTCCAGTCCAAT**AAATCCTCCCAAAAGTGAgtaccaagaaccacctcaagagtttgtgcagtttctatctccttattgtttttgtcttgggttgttatctgcaactctttgttgtaatactttggaattgtgtaccaataactgacacaagtctagtttatgtccttaagtttcctttatatatcaacatcttcaacacattctgatgttactatccgccatatataatcggagaagtacatgaaactacttttgcttatacacccatttgtgcatagaactaatgaaataatctccaaattctttgttcctctattctagttaatggagtaaaacacaaaccatagttatactctttttgataaagaagtacagttatggaataaatgaccaaaatctaaatatgccattaaatcaaagaga

***zwint1.1-1***

***3106 bp***

deleted sequence

tgagggaacatactcactgatatataattatgattttgattaaatttaattaagttttttttttctttttttttaaaaggggaagtaaacgttacagattaaaattaaagagtcttctggttcaatacgttacgattggtcataataaattgaaccggaattgcaattaacttcaaccctctgggtcaaattgattcaaatagaattcaggggttaatgttgaagaaaagtataaacctcggggagagttatgtttctcttccgtttctctttcacctttcccatttgaaaattgaaatcgtcttctctgctttcaacttcacaactacactctcttcagttctctaacagtagcgccaaaatgaatccccaagactaaagctttcttcaATGGAAGAAGAAACCCACGACGGATCTCTCGATCTCCAAGAGATTCGCAGgttttctacttctctttcgtctctttctttcattgatttttgtgctgctttgttcatctatttgttctcatttttctcagGCGCGTGAAAGAGCTCGATTTCTTTCCCCGGAATTGCAGAGAAGAGCCTGTAGAATCGTGTAGTTCCGATTACGAGACTCTGGTGGTTCAAGATTTTGTTCTTCAGTTTGAAgtaattgcgagatttcgagaacccttttccgtttttcaaatttgtctaattttcgattagattgctaatttcgttttcgtgtagCCAAAGGTGAAAGAGATTGTTGAAGAATACGGCGATGTTGATTTATTGGATGTTGAAGATTCAGgtttgctctgttgtttcctgtgagattaagatgaagtatgaaagtcaaataatggtgaaattaagtgatggaaatctgacagATGCTTACTTGGAGTACTTGAGAAATGAGCTTCAGTCTGTGGAGGCTGAAAGTGCCAAAGTTTCTGAAGAAATTGAACGTCTTTCTCAGTCTCATGCCCAAGgttcttcattagttttcttctactgatttagttttagatcatatagagtttgctactgttatttttataatatgagttttcttctatggatttgttagATTCTAGTAGGTTGCAAAGGGATCTTGAAGGTCTTTTACTGTCACTGGATTCTATGTCATCTCAGgttatttggttgaatagattatttctttggctggaatcaaaaggcttagttttgagagttatgtgattcttattttagGATGTGGAGAAGTCAAAAGAGAATCAACCATCTAGCAGCTCAATGGAAGTATGTGAAGTGATTGATGATGACAAGTTTAAGgtgccatgtttttaagttttgtttctaagagaagaagcttttgtgtgtgtgtgtgtgtgtgtgtgaaacatgtattaacaaaatttcatctatcttccagttattgattagagctaatgtggcagaatgtactaacctacttttcccgagcacgctaaggcagaagatcatatactgtcctttcagttataatttctaaataatttcgtagtggttaagacttaacatgaaaaagcatctattgtactctaaaatttccagatgttttttctagtacttactttctttgcttgtcactgcctatcttcttattgcttttatattgtgcatggatttgatcatctttcacatgtttttcagATGTTTGAACTCGAAAATCAGATGGAGGAGAAAAGGATGATTCTTAAGTCATTGGAAGATCTGGATTCGTTACGTAAAAGgtaatttaaacgtactttcagatttacatatttaccctgtcttcattagagttcttcacttgttctaccttcagGTTTGATGCTGCAGAACAAGTTGAGGACGCATTGACAGGCTTGAAGGTGCTCGAGTTTGACGGAAACTTTATTAGGCTCCAACTGCGAACATATATTCAAAAACTAGACGGTTTTCTTGGACAGCACAAATTTGACCACATTACTGAGCCATCTGAATTGATCCATGAATTGCTAATATACCTTAAGGATAAAACTACAGAGATAACAAAATTTGAGgtaagaacactctccgtctttcactgctgactattatgctttagttttgcataaatcctagataaatgtatctgtttcttgcttcttgttcctttttgtgaataaaattgaaatctgctttcttgctcagATGTTTCCAAATGATATATACATAGGAGACATCATCGAGGCAGCTGATTCTTTCAGgtttgcgctctagtatctttatgtcctgaaatttgtattacttatcattctgaaactgtatggtacgtaagttataagactaataagttatcagggacgataagatgatatcattctattattcttaaaactgcagGCAGGTAAGGTTACACTCTGCAGTGCTAGACACAAGATCTTCAGTCCAGTGGGTTGTCGCCAAAGTGCAAGATAAAATTATTTCAACAACTTTGAGAAAATATATTGTGATGAGTTCGAAAACAATTAGgtgagccttccctttcagtagcttgagaggctgaatttttctttcgtagctaactcatgtcattgagtgtctgagcaagccaattgactgcatgttttgtgatcctatggcagGTACACGTTTGAATACTACGACAAAGATGAAACAATTGTGGCTCATATAGCTGGAGGTATTGATGCATTTTTAAAGGTCTCTGATGGTTGGCCACTGCTGAATACCCCATTGAAGCTTGCATCTCTCAAGAACTCTGACAATCAGTCAAAGGGAATTTCTCTGAGCCTTATCTGCAAAGTTGAGgtaagatcatgtttctccaaactcaacactttcttctcgattttacaattccatcataaacaaaggtcttctctgttccttctcctggtcattgttgtcagtcttatttaatctctcaaatcgaacatttaggatatataataacatgattaagccattgtcctagaccaaatacagtttgcaacattgtagatgtaagtgcgtttggttttgctaagcgtaacgatttgtctcttgctctactcgtcagGAACTAGCGAATTCCTTGGATTTAGAGACCCGGCAAAACTTATCAGGCTTCATGGATGCAATTGAGAAAATACTCGTGGAGCAAACCCGTG

***zwint1.1-2***

***2390 bp***

Deleted sequence

tgagggaacatactcactgatatataattatgattttgattaaatttaattaagttttttttttctttttttttaaaaggggaagtaaacgttacagattaaaattaaagagtcttctggttcaatacgttacgattggtcataataaattgaaccggaattgcaattaacttcaaccctctgggtcaaattgattcaaatagaattcaggggttaatgttgaagaaaagtataaacctcggggagagttatgtttctcttccgtttctctttcacctttcccatttgaaaattgaaatcgtcttctctgctttcaacttcacaactacactctcttcagttctctaacagtagcgccaaaatgaatccccaagactaaagctttcttcaATGGAAGAAGAAACCCACGACGGATCTCTCGATCTCCAAGAGATTCGCAGgttttctacttctctttcgtctctttctttcattgatttttgtgctgctttgttcatctatttgttctcatttttctcagGCGCGTGAAAGAGCTCGATTTCTTTCCCCGGAATTGCAGAGAAGAGCCTGTAGAATCGTGTAGTTCCGATTACGAGACTCTGGTGGTTCAAGATTTTGTTCTTCAGTTTGAAgtaattgcgagatttcgagaacccttttccgtttttcaaatttgtctaattttcgattagattgctaatttcgttttcgtgtagCCAAAGGTGAAAGAGATTGTTGAAGAATACGGCGATGTTGATTTATTGGATGTTGAAGATTCAGgtttgctctgttgtttcctgtgagattaagatgaagtatgaaagtcaaataatggtgaaattaagtgatggaaatctgacagATGCTTACTTGGAGTACTTGAGAAATGAGCTTCAGTCTGTGGAGGCTGAAAGTGCCAAAGTTTCTGAAGAAATTGAACGTCTTTCTCAGTCTCATGCCCAAGgttcttcattagttttcttctactgatttagttttagatcatatagagtttgctactgttatttttataatatgagttttcttctatggatttgttagATTCTAGTAGGTTGCAAAGGGATCTTGAAGGTCTTTTACTGTCACTGGATTCTATGTCATCTCAGgttatttggttgaatagattatttctttggctggaatcaaaaggcttagttttgagagttatgtgattcttattttagGATGTGGAGAAGTCAAAAGAGAATCAACCATCTAGCAGCTCAATGGAAGTATGTGAAGTGATTGATGATGACAAGTTTAAGgtgccatgtttttaagttttgtttctaagagaagaagcttttgtgtgtgtgtgtgtgtgtgtgtgaaacatgtattaacaaaatttcatctatcttccagttattgattagagctaatgtggcagaatgtactaacctacttttcccgagcacgctaaggcagaagatcatatactgtcctttcagttataatttctaaataatttcgtagtggttaagacttaacatgaaaaagcatctattgtactctaaaatttccagatgttttttctagtacttactttctttgcttgtcactgcctatcttcttattgcttttatattgtgcatggatttgatcatctttcacatgtttttcagATGTTTGAACTCGAAAATCAGATGGAGGAGAAAAGGATGATTCTTAAGTCATTGGAAGATCTGGATTCGTTACGTAAAAGgtaatttaaacgtactttcagatttacatatttaccctgtcttcattagagttcttcacttgttctaccttcagGTTTGATGCTGCAGAACAAGTTGAGGACGCATTGACAGGCTTGAAGGTGCTCGAGTTTGACGGAAACTTTATTAGGCTCCAACTGCGAACATATATTCAAAAACTAGACGGTTTTCTTGGACAGCACAAATTTGACCACATTACTGAGCCATCTGAATTGATCCATGAATTGCTAATATACCTTAAGGATAAAACTACAGAGATAACAAAATTTGAGgtaagaacactctccgtctttcactgctgactattatgctttagttttgcataaatcctagataaatgtatctgtttcttgcttcttgttcctttttgtgaataaaattgaaatctgctttcttgctcagATGTTTCCAAATGATATATACATAGGAGACATCATCGAGGCAGCTGATTCTTTCAGgtttgcgctctagtatctttatgtcctgaaatttgtattacttatcattctgaaactgtatggtacgtaagttataagactaataagttatcagggacgataagatgatatcattctattattcttaaaactgcagGCAGGTAAGGTTACACTCTGCAGTGCTAGACACAAGATCTTCAGTCCAGTG

*zwint1.1-3*

2149 bp

Deleted sequence

ttgaaatcgtcttctctgctttcaacttcacaactacactctcttcagttctctaacagtagcgccaaaatgaatccccaagactaaagctttcttcaATGGAAGAAGAAACCCACGACGGATCTCTCGATCTCCAAGAGATTCGCAGgttttctacttctctttcgtctctttctttcattgatttttgtgctgctttgttcatctatttgttctcatttttctcagGCGCGTGAAAGAGCTCGATTTCTTTCCCCGGAATTGCAGAGAAGAGCCTGTAGAATCGTGTAGTTCCGATTACGAGACTCTGGTGGTTCAAGATTTTGTTCTTCAGTTTGAAgtaattgcgagatttcgagaacccttttccgtttttcaaatttgtctaattttcgattagattgctaatttcgttttcgtgtagCCAAAGGTGAAAGAGATTGTTGAAGAATACGGCGATGTTGATTTATTGGATGTTGAAGATTCAGgtttgctctgttgtttcctgtgagattaagatgaagtatgaaagtcaaataatggtgaaattaagtgatggaaatctgacagATGCTTACTTGGAGTACTTGAGAAATGAGCTTCAGTCTGTGGAGGCTGAAAGTGCCAAAGTTTCTGAAGAAATTGAACGTCTTTCTCAGTCTCATGCCCAAGgttcttcattagttttcttctactgatttagttttagatcatatagagtttgctactgttatttttataatatgagttttcttctatggatttgttagATTCTAGTAGGTTGCAAAGGGATCTTGAAGGTCTTTTACTGTCACTGGATTCTATGTCATCTCAGgttatttggttgaatagattatttctttggctggaatcaaaaggcttagttttgagagttatgtgattcttattttagGATGTGGAGAAGTCAAAAGAGAATCAACCATCTAGCAGCTCAATGGAAGTATGTGAAGTGATTGATGATGACAAGTTTAAGgtgccatgtttttaagttttgtttctaagagaagaagcttttgtgtgtgtgtgtgtgtgtgtgtgaaacatgtattaacaaaatttcatctatcttccagttattgattagagctaatgtggcagaatgtactaacctacttttcccgagcacgctaaggcagaagatcatatactgtcctttcagttataatttctaaataatttcgtagtggttaagacttaacatgaaaaagcatctattgtactctaaaatttccagatgttttttctagtacttactttctttgcttgtcactgcctatcttcttattgcttttatattgtgcatggatttgatcatctttcacatgtttttcagATGTTTGAACTCGAAAATCAGATGGAGGAGAAAAGGATGATTCTTAAGTCATTGGAAGATCTGGATTCGTTACGTAAAAGgtaatttaaacgtactttcagatttacatatttaccctgtcttcattagagttcttcacttgttctaccttcagGTTTGATGCTGCAGAACAAGTTGAGGACGCATTGACAGGCTTGAAGGTGCTCGAGTTTGACGGAAACTTTATTAGGCTCCAACTGCGAACATATATTCAAAAACTAGACGGTTTTCTTGGACAGCACAAATTTGACCACATTACTGAGCCATCTGAATTGATCCATGAATTGCTAATATACCTTAAGGATAAAACTACAGAGATAACAAAATTTGAGgtaagaacactctccgtctttcactgctgactattatgctttagttttgcataaatcctagataaatgtatctgtttcttgcttcttgttcctttttgtgaataaaattgaaatctgctttcttgctcagATGTTTCCAAATGATATATACATAGGAGACATCATCGAGGCAGCTGATTCTTTCAGgtttgcgctctagtatctttatgtcctgaaatttgtattacttatcattctgaaactgtatggtacgtaagttataagactaataagttatcagggacgataagatgatatcattctattattcttaaaactgcagGCAGGTAAGGTTACACTCTGCAGTGCTAGACACAAGATCTTCAGTCCAGTGGGTTGTCGCCAAAGTGCAAGATAAAATTATTTCAACAACTTTGAGAAAATA

*ZWINT1.2*

atatatatatatagttttaattaaaagttaatgttcgtggatcgatatgttaattcgttaggttttgtgaaacgaatctttaatatattttgtctgtagttttagcggatcggcccgaacggcccaaataatctcgaagcaccccatctgacatctctaaacatggttacaacttacttacaacatgtggcaaagctgtttaaagttacaaagtaacctaattagtaattacaaaatgaaaattgaaattgtttaaagtccggtacaatacttgaattgaatgcttcgattggtcaatccaaaattgaaccggaattgcaattaactcca**actctcgggggtctaattga**ttcaattataatttaggggttcaatgttgaagaaaattataaatctagggacgtttttcaaggttatgtatcttctcctttacctttcccatttgaaaattgaaatcgtcttctctgctttcaacttcacaactacactctcttcagttctctaacagtagcgccaaaatgaatccccaagactaaagctttcttcaATGGAAGAAGAAACCCACGACGGATCTCTCGATCTCCAAGAGATTCGCAGGttttctacttctctttcgtctctttctttcattgatttttgtgctgctttgttcatctatttgctctcatttttctcaggCGCGTGAAAGAGTTCGATTTCTTTCCCCGGAATTGCAGAGAAGAGCCTGTAGAATCGTGTAGTTCCGATTACGAGACTCTGGTGGTTCAAGATTTTGTTCTTCAGTTTGAAgtaattgcgagatttcgagaactcttttccgtttttcaaatttgtctaattttcgattagattgctaatttcgttttcgtgtagCCAAAGGTGAAAGAGATTGTTGAAGATTACGGCGATGTTGATTTATTGGATGTTGAtCACACACTTGTGttttgcagatgatttgttagttttcaccgatggcaagaaaagttctatagaaggaattcttcagatctttggtaaatttgcagatttctccggtttgcagattagtctcgagaagtcgaccatttacatggctggagtgaaagacaatgacaaagcagacattcttcacagtttcccttttgcctccg**gcgcactacctgttcgttac**ctagggctaccattgctgacaaaaaagatgacgacaagcgactacggccctctcgttgaaaaaatcagagtacgaataggtaaatggacggcgagacacctctcctttgcgggtcgtctacaactcattagttcggtaatccatagtctaaccaacttttggatgtctgcgtttcgtcttccaagtgcctgcatcaaagaaatcgatagcatttgctcttcttttctctggtctggccccgaactgaatacaaaaaaggctaaagtagcatggagtgatgtttgcacacccaaagatgaaggagggttaggcatcagatctttaaaggaagcaaacaaggtcagtttgttaaaactcatttggaggatgctctcgtctacttctttgtgggttcaatggttgcgattatatctgctgcgaaagggttccttttggtctatcagtgggaacacaactctaggatcatggatgtggaagaagattcttaaacacagagctttagcttcaggttttgtgaagcacgacattcacaatggcagcaatacatcgttttggtttgataactggtcaaagataggaaggctgattgatgtaacaggtcatcggggttgcattgacatgggaatcactcttcacgcctctgtagctgaagcggttgtgaaccatagaccaagacgccaccgacatgatactctgcttcgaatagaagacgtcattgccgaggtgcgccaccagggacttacttcgggggaggatacagttcgttggaagggaaatggggacatttttaaaccgtgttttaacacaaaggagacctgggctgcaactcgggaaccaaaactgaaagtcaactggtataagggtgtttggttttcacatgcaacgcctaaatactcagttttagcttggatagcgataaagaatcgtctaactacgggagatcggatgttaagttggaatgcgggagctgactcttcgtgtgtgctctgtcaccacctcgtggaaacgcgcgaccacttgttcttcacatgcccttactcagcagaggtatggtctacactcacacgaaagctcctttctcaacatttcaccaaccgctgggaagctattctaaagctcttaacaaacaagtcgttggggcacgaggttccgttcttgacaagatacacttttcagctaactctccactctctttggaaggaaagaaatggcagacgacatggagaagtgccgcaggctgcagctcaaatggttcgtttccttgataagcaagtccgaaataggatctcttccattcaaagccaagaagacagaagatataatggttgtatgacatgttggttcggttctagatagtgtggctctctacatattctttctgaccttattctttgactcttgtatctaaaatttataggcacaaacgttgcactcgatgtaaatcagttttttttcgaataaattttaaattttattcaaaaaaaaaaaaaagaagattcaggtttgctctgttgtttcctgtgagattaagatgaagtatgaaagtcaaataatggtgaaattaagtGATGGAAATCTGACAGATGCTTACTTGGAGTACTTGAGAAATGAGCTTCAGTCTGTGGAGGCTGAAAGTGCCAAAGTTTCTGAAGAAATTGAACGTCTTTCTCAGTCTCATGCCCTAGgttcttcattagttttcttctactgatttagttttagatcatatagagtttgctactgttatttttataatatgagttttcttctatggatttgttagATTCTAGTAGGTTGCAAAGGGATCTTGAAGGTCTTTTACTGTCACTGGATTCTATGTCATCTCAGgttatttggttgaatagattatttctttggctggaatcaaaaggcttagttttgagagttatgtgattcttattttagGATGTGGAGAAGTCAAAAGAGAATCAACCATCTAGCAGCTCAATGGAAGTATGTGAAGTGATTGATGATGACAAGTTTAAGgtgccatgtttttaagttttgtttctaagagaagaagcttttgtgtgtgtgtgtgtgtgaaacatgtattaacaaaattctttgcttgtcactgcctatcttcttattgcttttatattgtgcatggatttgatcatctttcacatgtttttcagATGTTTGAACTCGAAAATCAGATGGAGGAGAAAAGGATGATTCTTAAGTCATTGGAAGATCTGGATTCGTTACGTAAAaggtaatttaaacgtactttcagatttacatatttaccctgtcttcattagagttcttcacttgttctaccttcAGGTTTGATGCTGCAGAACAAGTTGAGGACGCATTGACAGGCTTGAAGGTGCTCGAGTTTGACGGAAACTTTATTAGGCTCCAACTGCGAACATATATTCAAAAACTAGACGGTTTTCTTGGACAGCACAAATTTGACCACATTACTGAGCCATCTGAATTGATCCATGAATTGCTAATATACCTGAAGGATAAAACTACAGAGATAACAAAATTTGAGgtaagaacactctccgtctttcactgctgactattatgctttagttttgcataaatcctagataaatgtatctgtttcttgcttcttgttcctttttgtgaataaaattgaaatctgctttcttgctcagATGTTTCCAAATGATATATACATAGGAGACATCATCGAGGCAGCTGATTCTTTCaggtttgcgctctagtatctttatgtcctgaaatttgtattacttatcattctgaaactgtatggtacgtaagttataagactaataagttatcagggacgataagatgatatcattctattattcttaaaactgcAGGCAGGTAAGGTTACACTCTGCAGTGCTAGACACAAGATCTTCAGTCCAGTGGGTTGTCGCCAAAGTGCAAGATAAAATTATTTCAACAACTTTGAGAAAAGATTTTGTGATGAGTTCGAAAACAATTaggtgagccttccctttcagtagcttgagaggctgaatttttctttcgtagctaactcatgtcattgagtgtctgagcaagccaattgactgcatgttttgtgatcctatggcAGGTACACGTTTGAATACTACGACAAAGATGAAACAATTGTGGCTCATATAGCTGGAGGTATTGATGCATTTTTAAAGGTCTCTGATGGTTGGCCACTGCTGAATACCCCATTGAAGCTTGCATCTCTCAAGAACTCTGACAATCAGTCAAAGGGATTTTCTCTGAGCCTTATCAGCAAACTTGAGgtaagatcatgtttctccaaactcaacactttcttctcgattttacaattccatcataaacaaaggtcttctctgttccttctccaggtcattgttgtcagtcttatttaatctctcaaatcgaacatttaggatatgtaataacatgattaagccattgtcctagaccaaatacagtttgcaacattgtagatgtaagtgcttttggttttgctcagcgtaacgatttgtctcttgctctactagtcagGAACTAGCGAACTCCTTGGATTTAGAGACCCGGCAAAACTTATCAGGCTTCATGGATGCTGTTGAGAAAATACTCGTGCAGCAAACCCGTGAAGAACTCAAGTCCAATGAATCCTCCCAAAAGTGAgtaccaagaaccacctcaagagtttgtgcagtttctatctccttattgtttttgtcttgggttgttatctgcaactctttgttgtaattactttggaaattggaattgtatcaatactcttgcttatgtcctcaagtttcctttatatatcaatatccttaaacacattattatcttactctccgccatatataatcagagaactaatgaaataatctccaaataatctccaaatact**tctccattctggttaatgg**aggaaaacacca**accatatataacttacggag**aagtaatctccaaataatctccaaatact**tctccattctggttaatg*g***aggaaaacaccaaccatatataacttagtgattacttctccgtaaggtgacaacaaaactcagaggtctctttaaatggaagaagctaaagttgttctttgagtgttttaactgttactttagtcaatttaaggaagtcaatatggcttagtaaatcaattaagaaaaccatttaaaatctaccaagttt

***zwint1.2-3 , zwint1.2-4, zwint1.2-4, zwint1.2-5, zwint1.2-6***

all have same 4807 bp deletion:

**ctcgggggtctaattga**ttcaattataatttaggggttcaatgttgaagaaaattataaatctagggacgtttttcaaggttatgtatcttctcctttacctttcccatttgaaaattgaaatcgtcttctctgctttcaacttcacaactacactctcttcagttctctaacagtagcgccaaaatgaatccccaagactaaagctttcttcaATGGAAGAAGAAACCCACGACGGATCTCTCGATCTCCAAGAGATTCGCAGGttttctacttctctttcgtctctttctttcattgatttttgtgctgctttgttcatctatttgctctcatttttctcaggCGCGTGAAAGAGTTCGATTTCTTTCCCCGGAATTGCAGAGAAGAGCCTGTAGAATCGTGTAGTTCCGATTACGAGACTCTGGTGGTTCAAGATTTTGTTCTTCAGTTTGAAgtaattgcgagatttcgagaactcttttccgtttttcaaatttgtctaattttcgattagattgctaatttcgttttcgtgtagCCAAAGGTGAAAGAGATTGTTGAAGATTACGGCGATGTTGATTTATTGGATGTTGAtCACACACTTGTGttttgcagatgatttgttagttttcaccgatggcaagaaaagttctatagaaggaattcttcagatctttggtaaatttgcagatttctccggtttgcagattagtctcgagaagtcgaccatttacatggctggagtgaaagacaatgacaaagcagacattcttcacagtttcccttttgcctccg**gcgcactacctgttcgttac**ctagggctaccattgctgacaaaaaagatgacgacaagcgactacggccctctcgttgaaaaaatcagagtacgaataggtaaatggacggcgagacacctctcctttgcgggtcgtctacaactcattagttcggtaatccatagtctaaccaacttttggatgtctgcgtttcgtcttccaagtgcctgcatcaaagaaatcgatagcatttgctcttcttttctctggtctggccccgaactgaatacaaaaaaggctaaagtagcatggagtgatgtttgcacacccaaagatgaaggagggttaggcatcagatctttaaaggaagcaaacaaggtcagtttgttaaaactcatttggaggatgctctcgtctacttctttgtgggttcaatggttgcgattatatctgctgcgaaagggttccttttggtctatcagtgggaacacaactctaggatcatggatgtggaagaagattcttaaacacagagctttagcttcaggttttgtgaagcacgacattcacaatggcagcaatacatcgttttggtttgataactggtcaaagataggaaggctgattgatgtaacaggtcatcggggttgcattgacatgggaatcactcttcacgcctctgtagctgaagcggttgtgaaccatagaccaagacgccaccgacatgatactctgcttcgaatagaagacgtcattgccgaggtgcgccaccagggacttacttcgggggaggatacagttcgttggaagggaaatggggacatttttaaaccgtgttttaacacaaaggagacctgggctgcaactcgggaaccaaaactgaaagtcaactggtataagggtgtttggttttcacatgcaacgcctaaatactcagttttagcttggatagcgataaagaatcgtctaactacgggagatcggatgttaagttggaatgcgggagctgactcttcgtgtgtgctctgtcaccacctcgtggaaacgcgcgaccacttgttcttcacatgcccttactcagcagaggtatggtctacactcacacgaaagctcctttctcaacatttcaccaaccgctgggaagctattctaaagctcttaacaaacaagtcgttggggcacgaggttccgttcttgacaagatacacttttcagctaactctccactctctttggaaggaaagaaatggcagacgacatggagaagtgccgcaggctgcagctcaaatggttcgtttccttgataagcaagtccgaaataggatctcttccattcaaagccaagaagacagaagatataatggttgtatgacatgttggttcggttctagatagtgtggctctctacatattctttctgaccttattctttgactcttgtatctaaaatttataggcacaaacgttgcactcgatgtaaatcagttttttttcgaataaattttaaattttattcaaaaaaaaaaaaaagaagattcaggtttgctctgttgtttcctgtgagattaagatgaagtatgaaagtcaaataatggtgaaattaagtGATGGAAATCTGACAGATGCTTACTTGGAGTACTTGAGAAATGAGCTTCAGTCTGTGGAGGCTGAAAGTGCCAAAGTTTCTGAAGAAATTGAACGTCTTTCTCAGTCTCATGCCCTAGgttcttcattagttttcttctactgatttagttttagatcatatagagtttgctactgttatttttataatatgagttttcttctatggatttgttagATTCTAGTAGGTTGCAAAGGGATCTTGAAGGTCTTTTACTGTCACTGGATTCTATGTCATCTCAGgttatttggttgaatagattatttctttggctggaatcaaaaggcttagttttgagagttatgtgattcttattttagGATGTGGAGAAGTCAAAAGAGAATCAACCATCTAGCAGCTCAATGGAAGTATGTGAAGTGATTGATGATGACAAGTTTAAGgtgccatgtttttaagttttgtttctaagagaagaagcttttgtgtgtgtgtgtgtgtgaaacatgtattaacaaaattctttgcttgtcactgcctatcttcttattgcttttatattgtgcatggatttgatcatctttcacatgtttttcagATGTTTGAACTCGAAAATCAGATGGAGGAGAAAAGGATGATTCTTAAGTCATTGGAAGATCTGGATTCGTTACGTAAAaggtaatttaaacgtactttcagatttacatatttaccctgtcttcattagagttcttcacttgttctaccttcAGGTTTGATGCTGCAGAACAAGTTGAGGACGCATTGACAGGCTTGAAGGTGCTCGAGTTTGACGGAAACTTTATTAGGCTCCAACTGCGAACATATATTCAAAAACTAGACGGTTTTCTTGGACAGCACAAATTTGACCACATTACTGAGCCATCTGAATTGATCCATGAATTGCTAATATACCTGAAGGATAAAACTACAGAGATAACAAAATTTGAGgtaagaacactctccgtctttcactgctgactattatgctttagttttgcataaatcctagataaatgtatctgtttcttgcttcttgttcctttttgtgaataaaattgaaatctgctttcttgctcagATGTTTCCAAATGATATATACATAGGAGACATCATCGAGGCAGCTGATTCTTTCaggtttgcgctctagtatctttatgtcctgaaatttgtattacttatcattctgaaactgtatggtacgtaagttataagactaataagttatcagggacgataagatgatatcattctattattcttaaaactgcAGGCAGGTAAGGTTACACTCTGCAGTGCTAGACACAAGATCTTCAGTCCAGTGGGTTGTCGCCAAAGTGCAAGATAAAATTATTTCAACAACTTTGAGAAAAGATTTTGTGATGAGTTCGAAAACAATTaggtgagccttccctttcagtagcttgagaggctgaatttttctttcgtagctaactcatgtcattgagtgtctgagcaagccaattgactgcatgttttgtgatcctatggcAGGTACACGTTTGAATACTACGACAAAGATGAAACAATTGTGGCTCATATAGCTGGAGGTATTGATGCATTTTTAAAGGTCTCTGATGGTTGGCCACTGCTGAATACCCCATTGAAGCTTGCATCTCTCAAGAACTCTGACAATCAGTCAAAGGGATTTTCTCTGAGCCTTATCAGCAAACTTGAGgtaagatcatgtttctccaaactcaacactttcttctcgattttacaattccatcataaacaaaggtcttctctgttccttctccaggtcattgttgtcagtcttatttaatctctcaaatcgaacatttaggatatgtaataacatgattaagccattgtcctagaccaaatacagtttgcaacattgtagatgtaagtgcttttggttttgctcagcgtaacgatttgtctcttgctctactagtcagGAACTAGCGAACTCCTTGGATTTAGAGACCCGGCAAAACTTATCAGGCTTCATGGATGCTGTTGAGAAAATACTCGTGCAGCAAACCCGTGAAGAACTCAAGTCCAATGAATCCTCCCAAAAGTGAgtaccaagaaccacctcaagagtttgtgcagtttctatctccttattgtttttgtcttgggttgttatctgcaactctttgttgtaattactttggaaattggaattgtatcaatactcttgcttatgtcctcaagtttcctttatatatcaatatccttaaacacattattatcttactctccgccatatataatcagagaactaatgaaataatctccaaataatctccaaatact**tctccattctggttaatgg**aggaaaacacca**accatatataacttacggag**aagtaatctccaaataatctccaaatact**tctccattctggtta**
