# Supplementary material for: The composition and structure of the outer kinetochore KMN complex is conserved across kingdoms
Source: Commun Biol. 2025 Nov 7;8:1543. doi: 10.1038/s42003-025-09120-6 (PMC12595034; doi:10.1038/s42003-025-09120-6)
Supplement: Supplementary file 3 — Description of Additional Supplementary Materials [file 42003_2025_9120_MOESM3_ESM.pdf]

# Description of Additional Supplementary Files

**File name:** Supplementary Data 1

**Description:** AP-MS Protein Identification details. Related to tables 1-2

**File name:** Supplementary Data 2

**Description:** Seed count details. Related to figure 4

**File name:** Supplementary Data 3

**Description:** Gene and mutation sequences for DSN1, NSL1 and ZWINT1 .

**File name:** Supplementary Data 4

**Description:** Primers and guide RNAs used in this study

**File name:** Supplementary Data 5

**Description:** AlphaFold2 pTM and ipTM scores. Related to figure 6-7
